# Primary Urachal Squamous Cell Carcinoma: A Case Report

**DOI:** 10.7759/cureus.70604

**Published:** 2024-10-01

**Authors:** Keerthana Rajyakodi, Barathi Gunabooshanam, Muthu Subramanian Paramasivan Sivakami, Sandhya Sundaram

**Affiliations:** 1 Pathology, Sri Ramachandra Institute of Higher Education and Research, Chennai, IND

**Keywords:** abdominal mass, partial cystectomy, primary urachal carcinoma, squamous cell carcinoma, urachus, urinary bladder carcinoma

## Abstract

The urachus is a tubular vestigial remnant extending from the anterior dome of the bladder to the umbilicus. Carcinoma arising from it is uncommon but aggressive. Primary urachal carcinoma, an epithelial neoplasm, is one such abnormality which is rare and aggressive accounting for a very small portion of all bladder cancers.

We herein present a case of a 48-year-old woman admitted with complaints of abdominal pain and abdominal mass. The mass was fixed to the anterior abdominal wall. A radiological investigation revealed a mass involving the midline of the anterior abdominal wall in the infraumbilical region with irregular margins along the course of the urachal ligament with surrounding fat stranding, suggesting a possibility of urachal malignancy with local extension. An ultrasound-guided core needle biopsy showed squamous cell carcinoma. The tumor was removed surgically. Further histological examination showed primary moderately differentiated squamous cell carcinoma of the urachus with metastasis to regional lymph nodes, extending into anterior abdominal muscles and up to the mucosa of the ileum. These findings correspond to the stage IVA of the Sheldon staging system for urachal carcinoma.

Due to the rarity of urachal squamous cell carcinoma and limited research, definitive treatment guidelines are lacking. Current recommendations are based on small case series and lack a standardized staging system. Surgical intervention remains the cornerstone of treatment, often encompassing complete resection of the urachus, umbilicus, adjacent involved tissue with free margins, and potentially the bladder or regional lymph nodes to mitigate the risk of metastasis and local recurrence.

## Introduction

The urachus is an embryonic channel that allows urine to drain from the bladder to the allantois through the umbilicus during fetal life. It normally closes by birth, becomes fibrotic, and forms the median umbilical ligament [1,2]. Abnormalities during this physiological process may lead to diseases of varying significance.

Urachal mass lesions are abnormalities associated with the urachus that can be either benign or malignant, highlighting the importance of accurate evaluation. The assessment begins with a comprehensive clinical history and physical examination, focusing on symptoms such as abdominal pain, discomfort, hematuria, urinary tract infections, or umbilical discharge. Differential diagnoses for urachal lesions include urachal cysts, urachal sinuses, urachal diverticula, and urachal carcinoma, the last being rare but aggressive. Imaging studies such as ultrasound and CT scans are essential for distinguishing between cystic and solid lesions, while MRI may be utilized for more complex cases.

Primary urachal carcinoma, an epithelial neoplasm, is one such abnormality which is rare and aggressive accounting for <1% of all bladder cancers [3]. Urachal carcinoma is a malignant epithelial tumor originating from the remnants of the urachus, a fetal structure connecting the umbilicus to the bladder. The median age of presentation is 52-59 years, and such a tumor has a poorer prognosis than urinary bladder tumors [4]. A meta-analysis of registry data revealed that adenocarcinoma was the most common tumor type in urachal carcinoma, accounting for 86% of cases. Urothelial carcinoma and squamous cell carcinoma followed, representing 12% and 2% of cases, respectively. A total of 29 cases of urachal squamous cell carcinoma have been documented [5]. Non-glandular carcinomas can exhibit various growth patterns, including cystic, cavitary, or solid infiltrative growth. We herein report a very rare primary squamous cell carcinoma arising from the urachus.

The Sheldon system, established in 1984, remains the most widely employed staging system for urachal carcinoma. Additional staging systems proposed for this condition include the simplified Mayo system and the Ontario system. While the tumor, node, metastasis (TNM) system has been adopted for bladder cancer, it is not its primary intended use and may be less applicable to tumors originating outside the urinary bladder.

Currently, there are no routinely used predictive molecular markers available for urachal carcinoma. Molecular features of urachal carcinoma, such as microsatellite instability (MSI) and mutations in the rat sarcoma virus family gene (RAS), may inform therapeutic decisions [4]. In advanced stages of bladder cancer, chemotherapy regimens traditionally utilized for colorectal adenocarcinoma may provide clinical benefits. Although less frequent, targeted therapeutic approaches have been reported.

## Case presentation

A 48-year-old woman presented with a two-month history of lower abdominal pain and a one-month history of an abdominal mass. She reported no urinary symptoms, hematuria, or abnormal bowel habits. Three years ago, the patient underwent a hysterectomy with bilateral salpingo-oophorectomy due to abnormal uterine bleeding. A histological examination of the surgical specimen confirmed the presence of adenomyosis.

**Figure 1:**
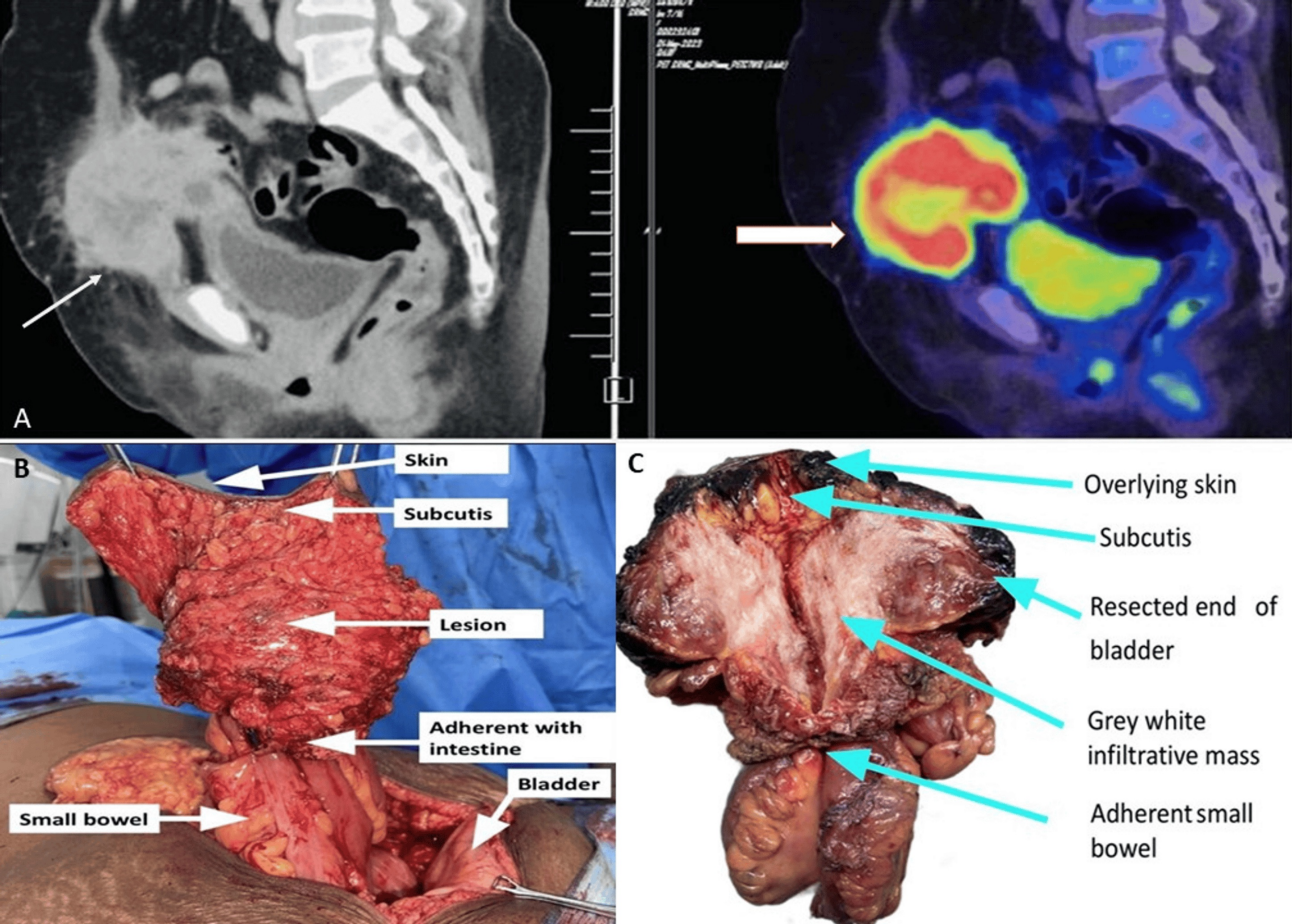
A) PET-CT image of a soft tissue mass. B) Intraoperative image of the mass. C) Serial sectioning of the mass showed a grey-white firm solid lesion. A) Both arrows pointing the heterogeneous, fluorodeoxyglucose (FDG)-avid soft tissue mass measuring approximately 70 x 76 x 90 mm (craniocaudal (CC) x anteroposterior (AP) x transverse (TR)) with irregular margins. B) Intraoperative image of the lesion adherent to the bowel wall and the abdominal wall. C) Cross-section of the mass demonstrates a grey-white firm solid irregular lesion measuring 12x8x8 cm. The mass was seen closely abutting the dome of the bladder and was seen adherent to the small bowel.

A physical examination revealed a 16-week-sized, lobulated, irregular, firm-to-hard mass measuring 10 x 12 cm in the hypogastric region, extending into both iliac fossae. The mass was fixed to the anterior abdominal wall, with no intrinsic mobility. The lower border of the mass was palpable at the level of the pubic symphysis. A positron emission tomography (PET)-CT scan revealed a heterogeneous, fluorodeoxyglucose (FDG)-avid soft tissue mass measuring approximately 70 x 76 x 90 mm (craniocaudal (CC) x anteroposterior (AP) x transverse (TR)) with irregular margins (Figure 1A). This mass was located in the midline of the anterior abdominal wall in the infraumbilical region, along the course of the urachal ligament, with surrounding fat stranding. The mass infiltrated the anterosuperior aspect of the urinary bladder wall. The PET scan also identified multiple enlarged lymph nodes in the external iliac and common iliac regions. No other FDG-avid lesions were detected elsewhere in the body.

The patient underwent an anterior abdominal wall mass excision, partial cystectomy, and bilateral pelvic node dissection, followed by anterior abdominal wall resection and reconstruction. During the procedure, a segment of the small bowel was found to be adherent to the lesion and was therefore excised. Bladder reconstruction was performed, and the surgical specimen was sent for a histological examination (Figure 1B, 1C).

A gross examination revealed a lobulated, partially skin-covered, predominantly fat-covered, firm mass measuring 15 x 15 x 15 cm. A segment of the small bowel measuring 16 cm in length was seen adherent with the lesion. A small portion of the urinary bladder dome was included for the examination (Figure 1B, 1C).

**Figure 2:**
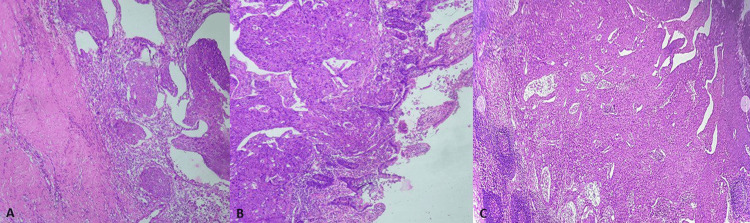
A) Tumor cells infiltrating the fibrous abdominal wall tissue (H&E stain 400X magnification). B) Tumor cells infiltrating small bowel mucosa (H&E stain 200X magnification). C) Metastatic deposits in the lymph nodes (H&E stain 200X magnification). H&E: hematoxylin and eosin

A histological examination confirmed the diagnosis of moderately differentiated squamous cell carcinoma arising from the urachus. Microscopically, the dome of the urinary bladder was free of disease (Figure 2A). However, neoplastic cells were observed infiltrating the small bowel wall, focally breaching the mucosa (Figure 2B). Metastatic deposits were also identified in the lymph nodes (Figure 2C).

**Figure 3:**
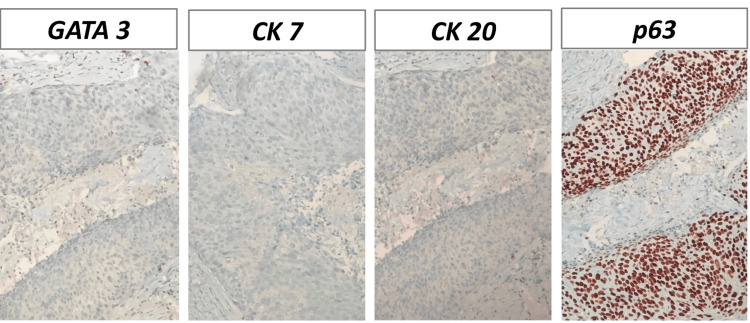
Immunohistochemical (IHC) staining was negative for GATA3, CK7, and CK20 but positive for p63 (IHC 400X magnification).

Immunohistochemical staining was negative for GATA3, CK7, and CK20 but positive for p63 (Figure 3). This immunohistochemical pattern further confirmed the primary origin of the tumor in the urachus.

## Discussion

The urachus is a closed vestigial musculo-fibrous remnant in humans located in the space of Retzius extending from the dome of the bladder to the umbilicus [1,5,6]. Failure of complete closure can in rare cases lead to numerous anomalies including fistula, diverticula, cysts, and sometimes even malignancy. Primary urachal carcinoma is a rare but aggressive entity mentioned first by Hue and Jacquin in 1863. Elaborate work was done by Cullen in 1916 and later it was categorized by C. Begg in 1930 [3,6-8].

Urachal carcinoma is a rare aggressive malignancy that accounts for about <1% of all bladder cancers [3]. It usually affects a younger population than bladder carcinoma, with a median age of 52-59 years [4,9]. Patients are often asymptomatic in the early stages and typically present at more advanced stages due to the lesion's natural tendency for early local invasion and distant metastasis [10].

Symptoms may arise if the tumor invades the bladder, with hematuria being the most frequent presentation in 90% of cases. Other possible symptoms include abdominal pain, recurrent urinary tract infections, or a palpable abdominal mass [11]. While various diagnostic criteria have been proposed, the criteria outlined by Sheldon et al. [12] are widely accepted among investigators.

Given the potential for urachal remnants to be present throughout the median umbilical ligament and the possibility of carcinoma arising within perivesical tissue, the staging of urachal carcinoma differs from that of bladder carcinoma.

The most widely used system is the Sheldon system from 1984, which classified tumors based on their extent of spread. Stage I is confined to the urachal mucosa (in situ). Stage II is a urachus invasion confined to the urachus itself. Stage III is a local extension to the bladder (IIIA), abdominal wall (IIIB), peritoneum (IIIC), or other viscera (IIID). Stage IV is metastasis to regional lymph nodes (IVA) or distant sites (IVB) [12-14].

The Mayo and Ontario staging systems, introduced in 2006, simplify the Sheldon staging system by utilizing a larger dataset. These systems reclassify bladder mucosa and muscularis propria invasion as stage II instead of stage III, and regional lymph node involvement as stage III instead of stage IVA. Distant metastasis remains classified as stage IV in both systems [15].

Later, a new set of criteria was introduced by the WHO in 2016 for the diagnosis of non-glandular urachal carcinomas, which are the following: a) tumor in bladder dome or anterior wall or midline supravesical to umbilicus, b) tumor epicenter away from the bladder surface, c) no primary tumor elsewhere of similar morphology, d) close relationship with urachal remnants, e) tumor does not involve the intact bladder surface, and f) if bladder surface involved, the tumor should have a reverse invasive front. The first three diagnostic criteria and any one of the last three criteria should be fulfilled [5,13].

A novel TNM staging system was proposed in 2023 based on a cohort of 626 cases extracted from the Surveillance, Epidemiology, and End Results (SEER) database. The proposed system categorizes urachal carcinoma into the following stages: stage Ip (T0, Ta, Tis), characterized by tumor confinement to the urachal mucosa without nodal involvement or distant metastasis; stage II (pT2, pT3a), encompassing tumors that invade the bladder muscularis propria or microscopically invade perivesical tissue; stage III (pT3b, pT4a), encompassing tumors that macroscopically invade bladder perivesical tissue or invade adjacent organs such as the uterus, vagina, or prostate; and stage IV (pT4b), defined by tumor invasion of the pelvic wall, abdominal wall, or peritoneum, with any nodal involvement or distant metastasis regardless of primary tumor extent [13,14].

Urachal carcinoma frequently metastasizes to distant sites, including the bones, lungs, liver, non-regional lymph nodes, and peritoneum. Despite these challenges, the mean five-year survival rate of approximately 50% surpasses that of stage-matched adenocarcinomas or urothelial carcinomas of the urinary bladder. Adverse prognostic indicators encompass a Sheldon tumor stage ≥ IIIB, a Mayo stage ≥ II, positive surgical margins, and the presence of lymph nodes or distant metastases [12-14]. While staging systems offer prognostic value for urachal carcinoma, their validation is limited by the relatively small sample sizes associated with the rarity of this disease.

The histological features of urachal carcinoma with squamous differentiation may exhibit both squamous and glandular components, while primary urachal squamous cell carcinoma is characterized solely by the squamous cell component.

The case presented here involved a midline tumor located along the urachal remnant structure, with its epicenter positioned away from the bladder surface. The tumor did not involve the bladder surface. Clinical and radiological evaluations confirmed the absence of other lesions and a negative history of malignant conditions, ruling out metastasis from other organs. The histological examination showed primary moderately differentiated squamous cell carcinoma of the urachus with metastasis to regional lymph nodes, extending into anterior abdominal muscles and up to the mucosa of the ileum. These findings correspond to the stage IVA of the Sheldon staging system for urachal carcinoma.

## Conclusions

In conclusion, although chemotherapy and radiation therapies have been used to treat urachal carcinoma, the effectiveness and safety of the therapies are unknown. Surgery is the mainstay option; complete removal of the urachus plus complete resection of the umbilicus and surrounding soft tissue is often done, usually combined with partial or complete cystectomy with nearby lymphadenectomy because metastasis and local recurrence are common. Primary urachal squamous cell carcinoma has a poor prognosis when compared to adenocarcinoma.
